# Taxonomic and nomenclatural notes on *Salix* (Salicaceae) from northern China

**DOI:** 10.3897/phytokeys.273.185575

**Published:** 2026-04-16

**Authors:** Zhenfeng Zhan, Hong Qu, Xin Zhang, Zhangjie Huang, Changli Zhao, Lei Wang

**Affiliations:** 1 School of Ecology and Nature Conservation, Beijing Forestry University, Beijing 100083, China College of Forestry, Northwest A&F University Yangling China; 2 Qinling National Forest Ecosystem Research Station, College of Forestry, Northwest A&F University, Yangling 712100, China Xishuangbanna Tropical Botanical Garden, Chinese Academy of Sciences Mengla China; 3 Xishuangbanna Tropical Botanical Garden, Chinese Academy of Sciences, Mengla 666303, China School of Ecology and Nature Conservation, Beijing Forestry University Beijing China; 4 National Forestry and Grassland Administration’s Scientific Research and Monitoring Base for Forests and Grasslands on the Qinghai-Tibet Plateau, Nyingchi City 860117, China College of Horticulture and Plant Protection, Henan University of Science and Technology Luoyang China; 5 College of Horticulture and Plant Protection, Henan University of Science and Technology, Luoyang 471000, China National Forestry and Grassland Administration’s Scientific Research and Monitoring Base for Forests and Grasslands on the Qinghai-Tibet Plateau Nyingchi City China

**Keywords:** Lectotype, new synonymy, northern China, taxonomic treatment, type specimen, willow

## Abstract

This study designates lectotypes for 20 names of *Salix* from northern China: Salix bikouensis var. villosa, *S. flavida*, *S. gordejevii*, *S. hsinganica*, *S. juparica*, S. matsudana var. anshanensis, *S. neomyrtillacea*, *S. pingliensis*, *S. pseudomatsudana*, *S. psammophila*, *S. qinlingica*, S. qinghaiensis var. microphylla, S. raddeana var. subglabra, *S. skvortsovii*, *S. subpyroliformis*, *S. sungkianica*, *S. tibetica*, *S. tschanbaischanica*, S. wilhelmsiana f. ciliuensis and *S. yanbianica*. We hypothesise that *S. babylonica* is actually an ancient cultivar derived from *S. matsudana* and we acknowledge the taxonomic status of both names. We propose a new synonymy of Salix matsudana var. anshanensis and S. matsudana var. pseudomatsudana under *S. matsudana*, as well as the synonymisation of S. wilhelmsiana f. ciliuensis under *S. cyanolimenaea*. For each name, the taxonomic background, current status and justification for lectotype designation and taxonomic treatment are provided in detail, supported by high-resolution images of the designated lectotypes.

## Introduction

The genus *Salix* (Salicaceae, Malpighiales) comprises 350–500 species worldwide, predominantly distributed across temperate and cold regions of the Northern Hemisphere, with only a few species occurring in the Southern Hemisphere (Skvortsov 1968, 1999; Argus 1997; Fang et al. 1999; Ohashi 2000; He et al. 2021; Belyaeva and King 2025). China is recognised as a diversity centre for *Salix* species (Skvortsov 1989; Ding 1995; Argus 1997). Records indicate that there are 275 willow species in China, amongst which 189 are endemic to the country (Fang et al. 1999).

Typification is a fundamental procedure in nomenclatural acts. It serves as the cornerstone of taxonomic practice, as it ensures the accuracy and stability of taxonomic names by providing an objective and verifiable material standard. Significant issues persist in the typification of Chinese *Salix* species. In recent years, studies on the lectotypification of Chinese *Salix* taxa have been successively published (He et al. 2019; Liu et al. 2020; Grabovskaya-Borodina et al. 2022).

During the period from 2020 to 2025, we conducted a comprehensive taxonomic revision of *Salix* in northern China. Throughout this study, we performed detailed examinations of the protologues and type specimens for a substantial number of taxa. We found that numerous taxonomic names of *Salix* exhibit ambiguity in their type designation, primarily due to four reasons. First, the holotype has been confirmed lost through research with no duplicates found (e.g. *S. psammophila*). According to Articles 9.3, 9.11 and 9.12 of the International Code of Nomenclature for algae, fungi and plants (Madrid Code, Turland et al. (2025)), a lectotype should be designated from the remaining type material. Second, the protologue fails to designate a type and only cites multiple gatherings (e.g. *S. gordejevii*), all of which constitute syntypes (Art. 9.6, Turland et al. (2025)). According to Article 9.3 (Turland et al. 2025), a lectotype may be chosen from these syntypes. Third, the protologue designates a gathering (generally with one collection number) as “type”, “typus”, “holotype” or “holotypus”, but research shows the gathering includes multiple duplicates and the author of the published name did not annotate any of these specimens as the type on the labels or merely marked all duplicates with the same type designation. (e.g. *S. skvortsovii*, *S. yanbianica*). Such designation fails to meet the necessary conditions for specifying a holotype, since a holotype must be a single specimen or a single illustration (Art. 9.1, Turland et al. (2025)). Consequently, these duplicates effectively constitute syntypes (Art. 9.6; Art. 40, N. 3, Ex. 3, Ex. 4, Turland et al. (2025)), from which a lectotype may be selected according to Article 9.3 (Turland et al. 2025). Given these circumstances and provided that no other scholars have previously designated lectotypes for these names, it is imperative to undertake lectotype designations for these taxonomic names. It should be noted that, based on the protologue and morphological characteristics of the type specimens, some type specimens clearly comprise multiple gatherings from different growth stages. The lectotype must be restricted to material from a single gathering within these specimens (Art. 8.1, Art. 8.2, Turland et al. (2025)).

Furthermore, to align the lectotype designations with current taxonomic concepts and to enhance the academic value of this study, it is necessary to clarify the nomenclatural background and current taxonomic status of these names. POWO (https://powo.science.kew.org/) aggregates the taxonomic views on *Salix* from sources such as Skvortsov (1968, 1999), Wang et al. (1984), Fang et al. (1999), Belyaeva (2014–, personal communication on Salicaceae) and Govaerts et al. (2021). Its classification framework for *Salix* provides the most recent reference. Therefore, the taxonomic status of most *Salix* names discussed in this study primarily follows the classification framework established by POWO. Additionally, our taxonomic views on a few names differ from those in POWO. In such cases, we have adopted classification perspectives proposed by other scholars or suggested our own interpretations of their taxonomic identity, adhering to the principle of priority (Art. 11.4, Turland et al. (2025)).

## Materials and methods

We conducted detailed studies of the protologues for all taxonomic names treated in this paper. Specimens deposited at the Herbaria BJFC, IFP, IGA, NEAU, NEFI, NENU, NWFC, PE, SDAU and WUK were examined in person. High-resolution images of type specimens housed at HIB, HNWP, LZD, NAS and KUN were examined via the Chinese Virtual Herbarium (CVH, http://www.cvh.ac.cn) and through loan requests by email. High-resolution images of type specimens deposited at A, E, K, LE, NY, P, S and US were also examined through the websites provided by NYBG (https://sweetgum.nybg.org/science/ih/) or via loan requests by email (herbarium codes follow Thiers (2025)). The taxonomic framework for *Salix* employed in this study primarily follows the classification perspectives of POWO. Typifications and taxonomic treatments of the names follow the International Code of Nomenclature for algae, fung, and plants (Turland et al. 2025). In evaluations of type status, specimen label data were critically examined and revised when necessary, using phenology to distinguish between type gatherings when the labels were not sufficiently informative.

## Results

### 1. Salix babylonica

Taxon classificationPlantaeAplousobranchiaPolycitoridae

L., Sp. Pl. 2: 1017. 1753

137C0185-0CB1-5E7F-8393-9A0E58C4CEA1

**Figure 1.**
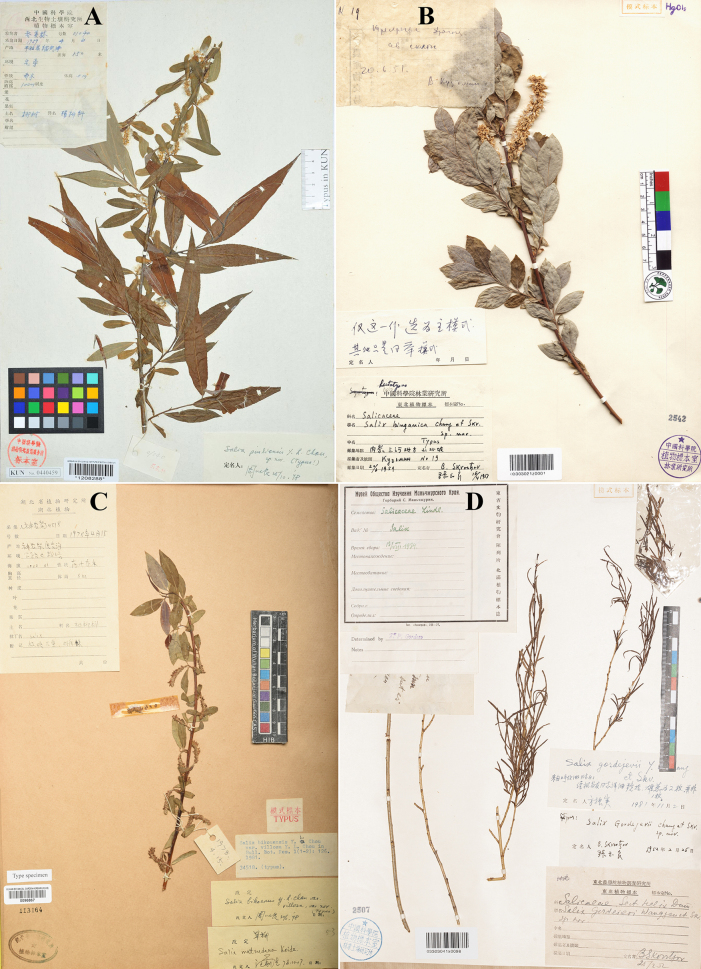
Type specimens of *Salix*. **A**. Lectotype of *S. pingliensis*; **B**. Lectotype of *S. hsinganica*; **C**. Lectotype of S. bikouensis var. villosa; **D**. Lectotype of *S. gordejevii* (photo credit: **A** KUN, available at https://www.cvh.ac.cn/; **B** IFP, available at https://www.cvh.ac.cn/, **C** by Guangwan Hu, **D** by Zhenfeng Zhan).

Salix babylonica L., Sp. Pl. 2: 1017. 1753. Type: Orient, Tournefort (lectotype designated by Jordaan (2005: 16): LINN1158.20, sterile, digital image examined).
= Salix pingliensis Y.L. Chou, Bull. Bot. Res., Harbin 1(1–2): 163. 1981. Type: China, Shanxi, Ping Li Xian, Mao Er Gou, 6 April 1957, *Z.L. Qiao 1040* (lectotype designated here: KUN barcode 1208288, ♀, digital image examined; isolectotypes: KUN barcode 1208287, digital image examined, PE barcode 00732723!, WUK barcode 0406761!) (Fig. 1A).

#### Note.

POWO treats *S. pingliensis* as a synonym of *S. babylonica*. We compared the type specimens of both and found no significant differences; therefore, this study agrees with the taxonomic treatment by POWO.

Chou (1981) described *S. pingliensis*, based on specimens collected from Pingli County, Shanxi. The protologue designated *Z.L. Qiao 1040* as the holotype and specified its deposition at KUN, but did not indicate a unique specimen identifier. Our investigation revealed that there are actually two specimens at KUN matching the designation: KUN1208288 and KUN1208287. Both specimens bear handwritten identification labels by Y.L. Chou with the notation “Typus”. Based on the available information, we cannot consider either specimen as the holotype; in effect, they collectively constitute syntypes. KUN1208288 has luxuriant branches and leaves, bearing numerous infructescences. Consequently, KUN1208288 is herein designated as the lectotype of *S. pingliensis*.

### 2. Salix bebbiana

Taxon classificationPlantaeAplousobranchiaPolycitoridae

Sarg., Gard. & Forest 8(404): 463. 1895.

4DC1F30A-B453-5572-9FAC-F3351BEA1606

Salix bebbiana Sarg., Gard. & Forest 8(404): 463. 1895. Type: Canada, Saskatchewan, *Richardson 13* (lectotype designated by Argus (1986: 91), K barcode 001079465, ♀ & sterile, digital image examined).
= Salix hsinganica Y.L. Chang & Skvortsov, Ill. Fl. Lign. Pl. N.-E. China: 173, 566. 1955. Type: China, Mongolia Interior borealis, regio San-che, 20 June 1951, *V. Kuzmin 19* (lectotype designated here: IFP barcode 03303021z0001!, ♀; isolectotype: NAS barcode 00280109, digital image examined) (Fig. 1B). Remaining syntypes: China, Mongolia, Interior borealis, prope Chailar, arenosis, 600 m alt., 10 June 1951, *Wang-Schang 1151* (n.v.); ibidem, *Wang-Schang 618* (IFP barcode 03303021x0066!, IFP barcode 03303021x0065!, ♀); ibidem, prope ostium fluminis Argum, in Monte Datsingou, inter Datsingou et Strelka, alt. 450 m, 5 August 1950, *A. Baranov & Y.C. Chu 323* (IFP barcode 03303022x0256!, IFP barcode 03303020x0226!, ♀); ibidem, regio oppida Chailar, in montibus, 700–800 m alt., 9 July 1951, *Wang-Schang 1198* (IFP barcode 03303021x0088!, ♀).

#### Note.

Y.L. Chang and Skvortsov described *S. hsinganica* in the Illustrated Manual of the Woody Plants of the Northeast Province, based on specimens collected from north-eastern Inner Mongolia (Skvortsov et al. 1955). This name was accepted by Wang et al. (1984) and Fang et al. (1999), though its relationship with *S. bebbiana* was not discussed. Comparison of the type specimens of both taxa revealed no significant morphological differences; instead, they consistently share key diagnostic characteristics, such as fruit stalk length and bract colour, indicating that they represent the same species of *Salix*. Therefore, this study concurs with Skvortsov (1968, 1999) in treating *S. hsinganica* as a synonym of *S. bebbiana*.

The protologue of *S. hsinganica* cited five gatherings: *V. Kuzmin 19*, *Wang-Schang 1151*, *Wang-Schang 618*, *A. Baranov & Y.C. Chu 323* and *Wang-Schang 1198*, but did not specify a specimen or gathering as the type (only stating that the type specimens were deposited at the IFP Herbarium). Clearly, all these gatherings constitute syntypes. During our examination of specimens at IFP, we located six specimens belonging to four of these gatherings. These specimens are in a similar condition, all possessing mature leaves and infructescences, along with the determination labels by Y.L. Chang. Consequently, IFP03303021z0001 is herein designated as the lectotype of *S. hsinganica*.

### 3. Salix bikouensis var. villosa

Taxon classificationPlantaeAplousobranchiaPolycitoridae

Y.L. Chou, Bull. Bot. Res., Harbin 1(1–2): 162. 1981.

CB3D389F-DB1F-56BC-B59B-FB683C4A784C

#### Type.

China, Hubei, Shennongjia, Tanghuanggou, 15 April 1978, *Plant Expedition Team 34518* (lectotype designated here: HIB barcode 0096867, ♀, digital image examined; isolectotype: HIB barcode 0096866, ♀, digital image examined) (Fig. 1C).

#### Note.

Chou (1981) described S. bikouensis var. villosa, based on specimens collected from Shennongjia, Hubei. This name is accepted in the current taxonomic system (https://powo.science.kew.org/). Chou (1981) designated *Plant Expedition Team 34518* (HIB) as the holotype of S. bikouensis var. villosa in the protologue. Our investigation revealed that there are two specimens at HIB that match the designation: HIB0096867 and HIB0096866. Both specimens are in a similar condition and bear the handwritten annotation “Typus” by Y.L. Chou. As it was impossible to conclusively identify a single specimen as the holotype, we herein designate HIB0096867 as the lectotype for S. bikouensis var. villosa.

### 4. Salix caprea

Taxon classificationPlantaeAplousobranchiaPolycitoridae

L., Sp. Pl. 2: 1020. 1753.

A89E0C5E-DE79-5BD5-AC99-F86223AE424F

Salix caprea L., Sp. Pl. 2: 1020. 1753. Type: Habitat in Europae siccis (lectotype designated by Jonsell and Jarvis (1994: 151): LINN1158.88, sterile, digital image examined).
= Salix raddeana var. subglabra Y.L. Chang & Skvortsov, Ill. Fl. Lign. Pl. N.-E. China: 170, 558. 1955. Type: China, Heilungkiang, Bai-mao-tzu et in Char-bin culta, 6 September 1952, *Wang-Schang 2305* (lectotype designated here: IFP barcode 03303019za001!, left-hand branch, sterile) (Fig. 5A). Remaining syntype: China, Heilungkiang, Bai-mao-tzu et in Char-bin culta, 10 May 1952, *Wang-Schang 2305* (IFP barcode 03303019za001!, right-hand branch, ♀); ibidem, *Wang-Schang 2245* (IFP barcode 03303029x0027, n.v.).

#### Note.

POWO treats S. raddeana var. subglabra as a synonym of *S. caprea*. We compared the type specimens of both and found no significant differences. Therefore, this study adopts the taxonomic treatment by POWO.

Y.L. Chang and Skvortsov described S. raddeana var. subglabra, based on specimen material collected from Harbin (Skvortsov et al. 1955). The protologue cited two gatherings, *Wang-Schang 2305* and *Wang-Schang 2245*, but did not specify a type (only stating that the type specimen was deposited at IFP). Therefore, both *Wang-Schang 2305* and *Wang-Schang 2245* constitute syntypes. Our investigation located two specimens matching this designation: IFP03303019za001 and IFP03303029x0027. We examined only IFP03303019za001, which is in good condition and bears a complete determination label annotated by Y.L. Chang as “var. nov.”. However, this specimen consists of a branch with mature leaves and a pistillate branch that are clearly from different collections, as our field studies in north-eastern China confirm that *S. caprea* flowers before leaf expansion; at the flowering stage, leaves are either not yet emerged or have just begun to sprout, making fully mature leaves impossible. Therefore, the lectotype must be restricted to one of these branches. Since the primary basis for the publication of this taxon was the indumentum on the abaxial leaf surface, the left branch with mature leaves on IFP03303019za001 is hereby designated as the lectotype for S. raddeana var. subglabra.

### 5. Salix cyanolimenaea

Taxon classificationPlantaeAplousobranchiaPolycitoridae

Hance, J. Bot. 20(238): 294. 1882.

94BFB100-F568-5488-80A0-38985E7EC201

Salix cyanolimenaea Hance, J. Bot. 20(238): 294. 1882. Type: China, Qinghai, Ko-ko-nor, 1881, *W. Mesny 22009* (BM barcode 000958033, ♀, digital image examined).
= Salix wilhelmsiana f. ciliuensis C.F. Fang & H.L. Yang, Fl. Desert. Reipubl. Popul. Sin. 1: 522, 280, 1985, syn. nov. Type: China, Gansu, Jinta, Chaohu, 10 May 1980, *C.F. Fang et al. 8027* (lectotype designated here: LZD barcode 0003723!, ♀; isolectotypes: LZD barcode 0003716!, LZD barcode 0003714!) (Fig. 5C).

#### Note.

POWO treats S. wilhelmsiana f. ciliuensis as a synonym of *S. wilhelmsiana*. By comparing the type specimens of S. wilhelmsiana f. ciliuensis, *S. wilhelmsiana* and *S. cyanolimenaea*, we found that the leaf width of S. wilhelmsiana f. ciliuensis more closely resembles that of the type specimen of *S. cyanolimenaea*. Therefore, this study disagrees with the treatment in POWO and instead considers S. wilhelmsiana f. ciliuensis as a synonym of *S. cyanolimenaea*.

C.F. Fang and H.L. Yang described S. wilhelmsiana f. ciliuensis in the Flora in desertis Reipublicae Populorum Sinarum, based on specimens collected from Jinta, Gansu (Yang 1985). The protologue designated *C.F. Fang et al. 8027* as the type and stated that the type specimen was deposited at the LZD Herbarium. Our investigation located three specimens matching this designation: LZD0003723, LZD0003716 and LZD0003714, all bearing complete identification labels by H.L. Yang and annotated with “f. nov.”, thus constituting syntypes. These three specimens are in a comparable state of preservation and each bears a complete determination label. Consequently, LZD0003723 is herein designated as the lectotype of S. wilhelmsiana f. ciliuensis.

### 6. Salix gordejevii

Taxon classificationPlantaeAplousobranchiaPolycitoridae

Y.L. Chang & Skvortsov, Ill. Fl. Lign. Pl. N.-E. China: 183, 553. 1955.

937D29EB-A931-53B3-ADBB-A7E5BAB689A0

**Figure 2.**
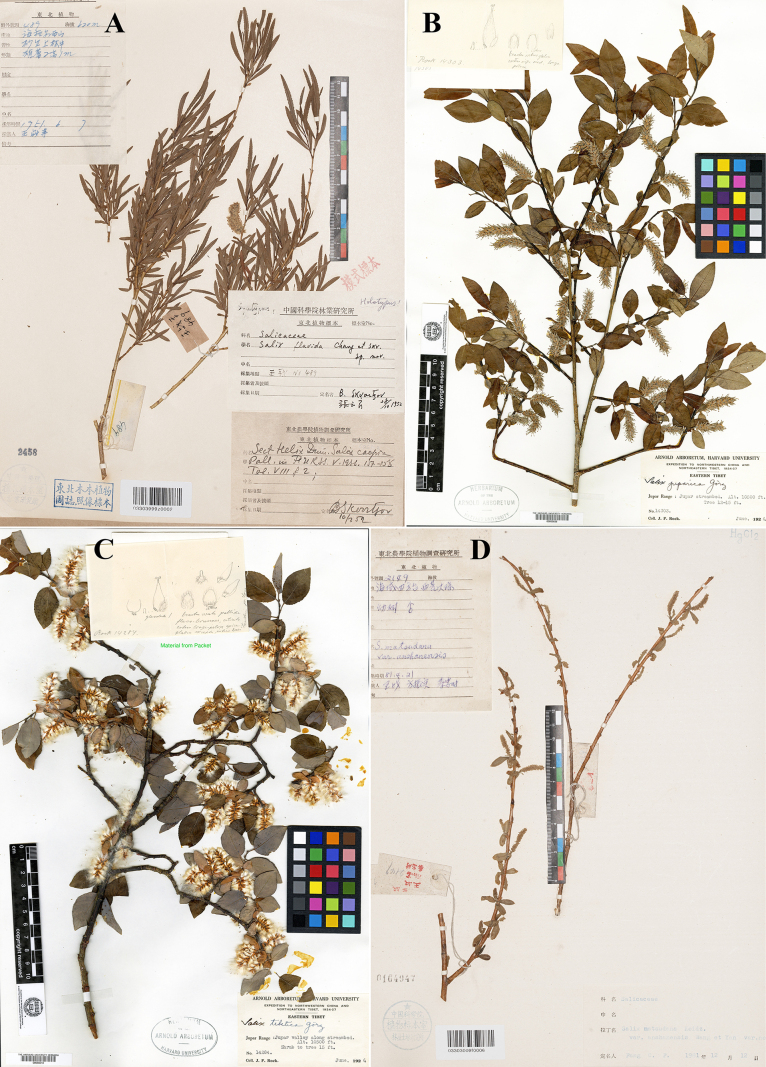
Type specimens of *Salix*. **A**. Lectotype of *S. flavida*; **B**. Lectotype of *S. juparica*; **C**. Lectotype of *S. tibetica*; **D**. Lectotype of Salix matsudana var. anshanensis (photo credit: **A, D** by Zhenfeng Zhan, **B**, **C** Herbarium of the Arnold Arboretum of Harvard University, available at https://kiki.huh.harvard.edu/databases/).

Salix gordejevii Y.L. Chang & Skvortsov, Ill. Fl. Lign. Pl. N.-E. China: 183, 553. 1955. Type: China, Mongolia Interior, prope Zagan nor, 13 August 1934, *T.P. Gordejev s.n*. (lectotype designated here: IFP barcode 03303041x0098!, ♂) (Fig. 1D). Remaining syntypes: China, Mongolia Interior, prope rivulum Khandagai gol, 10 August 1934, *T.P. Gordejev s.n*. (n.v.); ibidem, prope Chailar in arenosis, 5 August 1949, *M. Noda & Y.L. Chang s.n*. (n.v.); Prov. Je-che, Weng-nu-te, prope Chou-ke-tu in arenosis, 15 October 1951, *Wang-Kuan-cheng 239* (n.v.); prov. Liao-ning, Dschang-wu, prope Dschang-gu-tai in arenosis, 4 October 1952, *T.N. Liou 5461* (IFP barcode 03303041x0015!, IFP barcode 03303041x0014!, IFP barcode 03303041x0013!, IFP barcode 03303041x0010!, IFP barcode 03303041x0011!, IFP barcode 03303041x0012!, IFP barcode 03303041z0001!, sterile).
= Salix flavida Y.L. Chang & Skvortsov, Ill. Fl. Lign. Pl. N.-E. China: 167, 557. 1955. Type: China, Mongolia Interior, prope Chailar in arenosis, 7 June 1951, *Wang-Schang 489* (lectotype designated here: IFP barcode 03303999z0002!, ♂; isolectotypes: IFP barcode 03303041x0073!, IFP barcode 03303041x0072!, ♂) (Fig. 2A). Remaining syntypes: China, Liao-ning, Dschang-wu, prope Dschanggutai in arenosis, 4 October 1952, *T.N. Liou 5463* (syntype, IFP barcode 03303041x0016!, sterile); ibidem, 14 April 1952, *Wang-Schang 2190* (syntypes, IFP barcode 03303041x0053!, IFP barcode 03303041x0049!, IFP barcode 03303041x0050!, IFP barcode 03303041x0051!).

#### Note.

Y.L. Chang and Skvortsov described *S. gordejevii*, based on specimens collected by T.P. Gordejev near Hulun Lake, Inner Mongolia and by S.E. Liu near Zhanggutai, Liaoning (Skvortsov et al. 1955). This name is accepted in the current taxonomic system (https://powo.science.kew.org/). The protologue states that the stamens of this species are completely fused into a single unit. However, Wang et al. (1984) pointed out that the species actually possesses two distinct, free stamens. Through microscopic examination of the staminate type material collected by Gordejev near Hulun Lake, Inner Mongolia, deposited at IFP, we confirmed the presence of two separate stamens, indicating that the stamen description in the protologue is erroneous. A comparison of the type specimens of *S. flavida* and *S. gordejevii* reveals no significant morphological differences between them. Therefore, this study follows the current taxonomic view, recognising *S. gordejevii* and treating *S. flavida* as its synonym.

The protologue of *S. gordejevii* cited five gatherings: *T.P. Gordejev s.n*. (13 August 1934), *T.P. Gordejev s.n*. (10 August 1934), *M. Noda & Y.L. Chang s.n*., *Wang-Kuan-cheng 239* and *T.N. Liou 5461*, but did not designate a type (only stating that the type specimens were deposited at IFP). Clearly, all these gatherings constitute syntypes. During our examination of specimens at IFP, we located two of these gatherings: *T.P. Gordejev s.n*. (13 August 1934) and *T.N. Liou 5461*. The gathering *T.N. Liou 5461* is represented by seven specimens, all consisting of branches with mature leaves. The gathering *T.P. Gordejev s.n*. (13 August 1934) is represented by a single specimen (IFP03303041x0098), which possesses mature leaves and staminate inflorescences. IFP03303041x0098 bears the complete determination label annotated with “sp. nov.” by Y.L. Chang; and an additional determination label indicates that C.Y. Yang had previously conducted a detailed microscopic examination of its staminate inflorescences, confirming that the stamens are two in number and completely free (the free stamens are a key diagnostic character for this species). Therefore, IFP03303041x0098 is herein designated as the lectotype for *S. gordejevii*.

In describing *S. flavida*, Skvortsov et al. (1955) cited *Wang-Schang 489*, *T.N. Liou 5463* & *Wang-Schang 2190*, but did not designate a type (only stating that the type specimens were deposited at IFP). Clearly, *Wang-Schang 489*, *T.N. Liou 5463* and *Wang-Schang 2190* all constitute syntypes. *Wang-Schang 2190* was collected in the early flowering state; its inflorescences were not fully expanded and leaves had not yet emerged; *T.N. Liou 5463* is represented by only one specimen, collected at the end of the vegetative state with lax branches and leaves; *Wang-Schang 489* was collected in the early vegetative stage and is represented by three specimens, amongst which IFP03303999z0002 possesses well-preserved branches, leaves and a remnant staminate inflorescence. Consequently, IFP03303999z0002 is herein designated as the lectotype of *S. flavida*.

### 7. Salix juparica

Taxon classificationPlantaeAplousobranchiaPolycitoridae

Goerz, J. Arnold Arbor. 13(4): 391. 1932.

D698BDE7-886B-50EB-A72E-A5DE2BE1B70A

#### Type.

China, Qinghai, Jupar Range, 3150 m alt., June 1926, *J.F. Rock 14303* (lectotype designated here: A barcode 00055836, ♀, digital image examined; isolectotypes: S13-13584, US barcode 00105146, digital image examined) (Fig. 2B). Remaining syntypes: China, Qinghai, Jupar valley along streambed, 3150 m alt., June 1926, *J.F. Rock 14283* (A barcode 00055835, S barcode 13-13587, digital image examined); China, Radja and Yellow River, 3150 m alt., 2 June 1926, *J.F. Rock 14085* (A barcode 00055834, S13-13588, US barcode 01269107, digital image examined); ibidem, northern slopes of river valley mountains opposite Radja, 3150 m alt., 27 May 1926, *J.F. Rock 14001* (S13-13586, digital image examined).

#### Note.

Goerz (1932) described *S. juparica*, based on specimens collected from the Jupar Range, Qinghai. This name is accepted in the current taxonomic system (https://powo.science.kew.org/). The protologue cited four gatherings: *J.F. Rock 14303*, *J.F. Rock 14283*, *J.F. Rock 14085* and *J.F. Rock 14001*, but did not designate a type. Clearly, all these gatherings constitute syntypes. Syntypes of these four gatherings were located at A, S and US herbaria. Amongst them, A00055836 possesses numerous well-preserved mature leaves and young infructescences; furthermore, the accompanying illustration comprehensively displays the gynoecial morphology of this taxon. Therefore, A00055836 is herein designated as the lectotype of *S. juparica*.

### 8. Salix juparica var. tibetica

Taxon classificationPlantaeAplousobranchiaPolycitoridae

 (Goerz) C.F. Fang, Fl. Reipubl. Popularis Sin. 20(2): 232. 1984.

1F6B80BB-EA35-5DA1-B587-DC2F4D22733C

≡ Salix tibetica Goerz, J. Arnold Arbor. 13(4): 391. 1932. Type: China, Jupar Range, Jupar Valley along streambed, 3150 m alt., June 1926, *J.F. Rock 14284* (lectotype designated here: A barcode 00056018, ♀; isolectotypes: P barcode 00741179, S barcode 13-15778, US barcode 00105251, digital images examined) (Fig. 2C). Remaining syntypes: China, Jupar Range, Jupar Valley along streambed, 3150 m alt., June 1926, *J.F. Rock 14304* (A barcode 00056019, P barcode 00741180, S13-15777, US barcode 00105250, ♂, digital images examined).

#### Note.

Goerz (1932) described *S. tibetica*, based on the material collected from the Jupar Range, Qinghai. After examining the type specimens of both *S. juparica* and *S. tibetica*, Fang (1984) concluded that their only distinguishing character was the pubescent ovary in *S. juparica* versus the glabrous ovary in *S. tibetica*, which was insufficient to treat them as two distinct species. Consequently, Fang (1984) recombined *S. tibetica* as S. juparica var. tibetica, a treatment followed by Fang et al. (1999). This study adopts the treatment by Fang (1984) and opposes POWO’s treatment of S. juparica var. tibetica as a separate species, *S. tibetica*.

In the protologue of *S. tibetica*, Goerz (1932) cited two gatherings, *J.F. Rock 14284* and *J.F. Rock 14304*, but did not designate a single gathering as the type. Our investigation located multiple well-preserved specimens from these gatherings at A, P, S and US herbaria: A00056018, A00056019, P00741179, P00741180, S13-15777, S13-15778, US00105250 and US00105251. The pistillate flowers provide greater taxonomic value for delimiting this taxon compared to the staminate flowers; therefore, the lectotype should be preferably selected from female specimens. A00056018 includes a detailed illustration of a dissected pistillate flower, which comprehensively demonstrates the gynoecial morphology of this taxon. Thus, A00056018 is herein designated as the lectotype of *S. tibetica*.

### 9. Salix kangensis

Taxon classificationPlantaeAplousobranchiaPolycitoridae

Nakai, Bot. Mag. (Tokyo) 30(356): 275. 1916.

F37FF579-CEBC-5510-8DF3-BE8AF2D25BC3

Salix kangensis Nakai, Bot. Mag. (Tokyo) 30(356): 275. 1916. Type: Corea, Phyong-an, Kang-gei, *Mills 301* (TI, not seen).
= Salix skvortzovii Y.L. Chang & Y.L. Chou, Woody Pl. Xiao Hinggan Mts.: 86. 1955. Type: China, Heilungkiang, Dailing, 6 May 1953, *Chang Yu Liang 9* (lectotype designated here: NEAU barcode 033003040000003!, left-hand branch, ♀; isolectotypes: NEAU barcode 0009999!, NEAU barcode 0009998!, NEAU barcode 0009997!, NEAU barcode 0009993!, NEAU barcode 0009996!, NEAU barcode 0009992!, NEAU barcode 033003040000001!) (Fig. 5B).

#### Note.

POWO treats *S. skvortzovii* as a synonym of *S. kangensis*. We conducted a detailed comparison between the type specimen of *S. skvortzovii* and the protologue of *S. kangensis* and found no significant differences. Therefore, this study concurs with the taxonomic treatment by POWO.

Chang and Chou (1955) described *S. skvortsovii*, based on specimens collected from Dailing, Heilongjiang. The protologue designated *Chang Yu Liang 9* as the type and specified its deposition at NEAU. Our investigation located eight specimens of *Chang Yu Liang 9* at NEAU. These eight syntypes are in a comparable state of preservation. Amongst them, only NEAU033003040000003 and NEAU0009999 bear a handwritten determination label annotated with “Typus” by Y.L. Chang. However, the pistillate inflorescences on NEAU033003040000003 are in a superior state of preservation. Furthermore, NEAU033003040000003 consists of a branch with leaves and a pistillate flower branch. This species flowers before leaf expansion and leaves only begin to mature after the fruiting period has concluded. Based on our field observations of populations near Dailing (the type locality), the flowering period occurs from May to June. Therefore, it is impossible to observe fully mature leaves in a population collected on 6 May. Additionally, the leaves on this specimen are thick in texture and several show clear signs of damage, which typically occurs in the later vegetative stage. This indicates that the leafy branch and the pistillate flower branch on this specimen were collected at different times and belong to two separate gatherings. Since the protologue specifies the type collection date as the flowering period (6 May 1953), the left pistillate branch on specimen NEAU033003040000003 is hereby designated as the lectotype of *S. skvortsovii*.

### 10. Salix matsudana

Taxon classificationPlantaeAplousobranchiaPolycitoridae

Koidz., Bot. Mag. (Tokyo) 29: 312. 1915.

7AD06380-0CE7-5584-950D-F16EADCB0597

**Figure 3.**
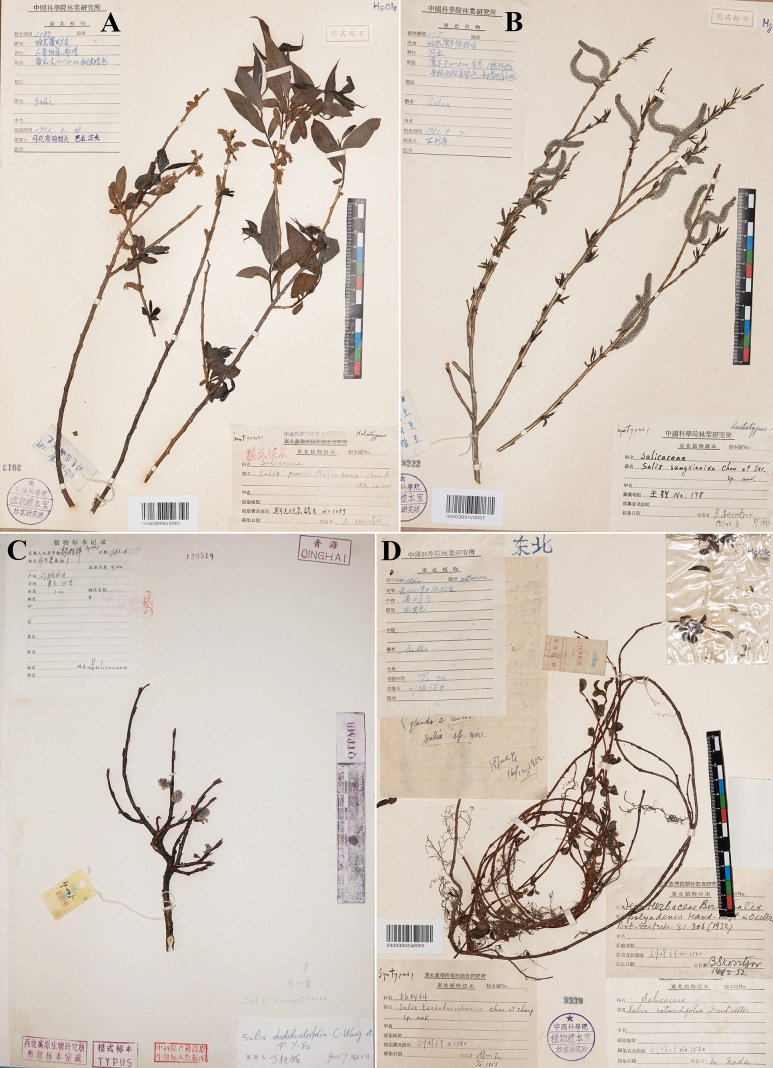
Type specimens of *Salix*. **A**. Lectotype of *S. pseudomatsudana*; **B**. Lectotype of *S. sungkianica*; **C**. Lectotype of *S. neomyrtillacea*; **D**. Lectotype of *S. tschanbaischanica* (photo credit: **A**, **B**, **D** by Zhenfeng Zhan, **C** HNWP, available at https://www.cvh.ac.cn/).

Salix matsudana Koidz., Bot. Mag. (Tokyo) 29: 312. 1915. Type: China, Kansu, Ranshiu, 17 April 1907, *Z. Umemura 17* (TI barcode 00083740, ♀ & ♂, digital image examined).
= Salix pseudomatsudana Y.L. Chou & Skvortsov, Ill. Fl. Lign. Pl. N.-E. China: 149, 552. 1955, syn. nov. ≡ Salix matsudana var. pseudomatsudana (Y.L. Chou & Skvortsov) Y.L. Chou, Fl. Reipubl. Popularis Sin. 20(2): 134. 1984. Type: China, Charbin, 28 May 1952, *B.V. Skvortsov 1089* (lectotype designated here: IFP barcode 03303999z0005!, ♀; isolectotypes: IFP barcode 03303009a0041!, IFP barcode 03303009a0042!, IFP barcode 03303009a0043!) (Fig. 3A). Remaining syntype: China, Charbin, *B.V. Skvortsov 1090* (IFP barcode 03303009a0040!, sterile).
= Salix matsudana var. anshanensis C. Wang & J.Z. Yan, Bull. Bot. Res., Harbin 1(1–2): 176. 1981, syn. nov. Type: China, Liaoning, Anshan Shi, Haicheng, Xihuangdi. *Wang Chan, Fang Cheng-fu, Qin Zhong-shi 2149* (lectotype designated here: IFP barcode 03303009f0006!, ♂; isolectotype: IFP barcode 03303009f0007!, ♂ & sterile) (Fig. 2D).

#### Note.

The taxonomic status of *S. matsudana* remains contentious. Wang et al. (1984), Fang et al. (1999) and Ohashi (2001) recognised this species, while Skvortsov (1968, 1999) treated it as a synonym of *S. babylonica*. Based on our research, *S. matsudana* and *S. babylonica* are morphologically very similar, with a significant number of intermediate forms between them. The presence or absence of a dorsal nectary in pistillate flowers and whether the branches are distinctly pendulous are insufficient to reliably distinguish these intermediate individuals. However, these two characters can clearly differentiate typical individuals. Our field investigations show that individuals in their natural state generally have non-pendulous branches and pistillate flowers that mostly possess a dorsal nectary, aligning more closely with *S. matsudana*. In contrast, the widely cultivated individuals with distinctly pendulous branches and pistillate flowers usually lacking a dorsal nectary match *S. babylonica*. Accordingly, we hypothesise that *S. babylonica* is actually an ancient cultivar derived from *S. matsudana* and retain both names as holding certain taxonomic value. In light of this ongoing debate, this study adopts the viewpoint of Wang et al. (1984) and others, accepting *S. matsudana*. POWO treats *S. matsudana*, Salix matsudana var. anshanensis and *S. pseudomatsudana* as synonyms of *S. babylonica*. Based on comparative studies of their protologues and type specimens, Salix matsudana var. anshanensis and *S. pseudomatsudana* are consistent with the overall morphological characteristics of *S. matsudana*. Since this study recognises *S. matsudana*, we here treat Salix matsudana var. anshanensis and *S. pseudomatsudana* as synonyms of *S. matsudana*.

The protologue of Salix matsudana var. anshanensis designated *Wang Chan, Fang Cheng-fu, Qin Zhong-shi 2149* (♂) as the type and specified that the type specimen was deposited at IFP. Our investigation located two specimens matching this designation: IFP03303009f0007 and IFP03303009f0006. Both specimens bear complete identification labels by C.F. Fang (IFP03303009f0007 has a handwritten label and IFP03303009f0006 has a printed label), but neither is explicitly marked as the type. Therefore, based on the available information, we cannot identify either specimen as the holotype; they effectively constitute syntypes. Since Yan et al. (1981) specified male material as the type, the lectotype should be selected from staminate branches. IFP03303009f0006 contains two well-preserved staminate branches, whereas the staminate branches on IFP03303009f0007 are poorly preserved. Consequently, IFP03303009f0006 is herein designated as the lectotype of Salix matsudana var. anshanensis.

The protologue of *S. pseudomatsudana* cited two gatherings: *B.V. Skvortsov 1089* and *B.V. Skvortsov 1090*, but did not designate a type (only stating that the type specimens were deposited at IFP). Our investigation located five specimens matching the designation: IFP03303999z0005, IFP03303009a0041, IFP03303009a0042, IFP03303009a0043 and IFP03303009a0040, all of which constitute syntypes. Amongst these, only IFP03303999z0005 possesses both infructescences and well-developed leaves and bears a handwritten determination label annotated with “sp. nov.” by Skvortsov. Consequently, IFP03303999z0005 is hereby designated as the lectotype of *S. pseudomatsudana*.

### 11. Salix miyabeana

Taxon classificationPlantaeAplousobranchiaPolycitoridae

Seemen, Bot. Jahrb. Syst. 21(4): 50. 1896.

50064816-84A6-519B-A081-310C7B45EF86

Salix miyabeana Seemen, Bot. Jahrb. Syst. 21(4): 50. 1896. Type: Japan, Sapporo, prov. Ishikari; April, May, October 1891, *Y. Tokubuchi s.n*. (SAP, not seen; B, ♀ and sterile, photo examined: Hao (1936: fig. 86)).
= Salix sungkianica Y.L. Chou & Skvortsov, Ill. Fl. Lign. Pl. N.-E. China: 181, 552. 1955. Type: China, Heilungkiang, Charbin, prope rivulum Madiagou, 7 May 1951, *Wang-Wei & Wang-Schang 178* (lectotype designated here: IFP barcode 03303031z0002, ♀; isolectotypes: IFP barcode 03303031x0087!, IFP barcode 03303031x0088!, IFP barcode 03303031x0089!, IFP barcode 03303031x0090!, IFP barcode 03303031x0091!, IFP barcode 03303031x0092!, IFP barcode 03303031x0093!, IFP barcode 03303031x0094!) (Fig. 3B). Remaining syntypes: China, Heilungkiang, Charbin, prope rivulum Madiagou, *Wang-Wei & Wang-Schang 186* (IFP barcode 03303031z0001!, IFP barcode 03303031x0112!, IFP barcode 03303031x0113!, IFP barcode 03303031x0115!, IFP barcode 03303031x0116!, IFP barcode 03303031x0117!, ♂); ibidem, *Wang-Wei & Wang-Schang 188* (IFP barcode 03303031x0121!, IFP barcode 03303031x0122!, IFP barcode 03303031x0123!, IFP barcode 03303031x0124!, IFP barcode 03303031x0125!, IFP barcode 03303031x0126!, IFP barcode 03303031x0127!, IFP barcode 03303031x0128!, IFP barcode 03303031x0129!, ♂); ibidem, 21 August 1952, *Wang-Wei & Wang-Schang 230* (IFP barcode 03303033x0260!, IFP barcode 03303033x0261!, IFP barcode 03303033x0262!, sterile); ibidem, *Wang-Wei & Wang-Schang 2234* (IFP barcode 03303031x0014!, ♀).

#### Note.

This species exhibits a very wide distribution range and considerable morphological variation, particularly in leaf shape, presence or absence of stipules, length of inflorescences and infructescences and style length, which has led to taxonomic confusion and controversy. Skvortsov (1968, 1999) conducted a comprehensive study of the relevant names and ultimately accepted *Salix miyabeana* Seemen as the correct name for this taxonomically problematic group. Based on our examination of original materials and numerous herbarium specimens, we consider that the morphological characteristics of *S. sungkianica* fall entirely within the range of variation of *S. miyabeana*, indicating that they represent the same species. Therefore, we agree with Skvortsov (1968, 1999) in treating *S. sungkianica* as a synonym of *S. miyabeana*, while opposing POWO’s treatment of *S. sungkianica* as an accepted name.

The protologue of *S. sungkianica* cited five gatherings: *Wang-Wei & Wang-Schang 178*, *Wang-Wei & Wang-Schang 186*, *Wang-Wei & Wang-Schang 188*, *Wang-Wei & Wang-Schang 230* and *Wang-Wei & Wang-Schang 2234*, but did not specify a type (only stating that the type specimens were deposited at IFP). We located 28 specimens at IFP matching this designation, which constitute syntypes. The species was primarily described, based on the morphology of pistillate flowers; therefore, the lectotype is to be preferably selected from the female syntypes (*Wang-Wei & Wang-Schang 178* and *Wang-Wei & Wang-Schang 2234*). IFP03303031z0002 is in excellent condition, possessing numerous fully developed pistillate inflorescences and young leaves and bears a handwritten determination label by Skvortsov. Consequently, IFP03303031z0002 is herein designated as the lectotype for *S. sungkianica*.

### 12. Salix myrtillacea

Taxon classificationPlantaeAplousobranchiaPolycitoridae

Andersson, J. Linn. Soc., Bot. 4: 51. 1860.

C3738A95-CACA-586F-9F92-016360658DBB

Salix myrtillacea Andersson, J. Linn. Soc., Bot. 4: 51. 1860. Type: India, Sikkim, Lachen, 3657 m alt., 20 June 1984, *J.D. Hooker s.n*. (syntypes, K barcode 000335051, A barcode 00031227, ♀ and sterile, digital images examined).
= Salix neomyrtillacea Ch.Y. Yang & Z.D. Wei in Li, Ligneous Flora of Qinghai: 131. 1987. Type: China, Qinghai, Nangqian Hsien, Mozhang, 4100 m alt., June 1982, *Z.D. Wei 005* (lectotype designated here, HNWP barcode 120519!, ♀; isolectotype: HNWP barcode 120518, ♀ only, digital image examined); ibidem, June 1982, *Z.D. Wei 004* (isolectotypes: HNWP barcode 120522, HNWP barcode 120521, HNWP barcode 120520, digital images examined) (Fig. 3C); ibidem, August 1982, *Z.D. Wei 061* (paratype, HNWP barcode 120518, sterile only, digital image examined).

#### Note.

Ch.Y. Yang and Z.D. Wei described *S. neomyrtillacea*, based on specimens collected from Nangqian Hsien, Qinghai (Wei 1987). The protologue states that this species is similar to *S. myrtillacea*, differing in its smaller leaves that are often entire-margined and its shorter styles, approximately one-third the length of the ovary. A comparison of the type specimens reveals that leaf length in *S. neomyrtillacea* ranges from 1.8–3 (3.5) cm, while that of *S. myrtillacea* is within 1.5–3 cm, indicating no significant difference in leaf size. Based on our field surveys of over ten populations in north-eastern Qinghai, central and southern Gansu and northern Sichuan, as well as examination of numerous herbarium specimens, *S. myrtillacea* exhibits considerable intraspecific variation. This variation includes plant height ranging from 0.5 to 5 m, leaf margins entire or distinctly glandular-serrate, mature leaves glabrous to distinctly pubescent and style length varying markedly, typically 0.3–0.8 mm, shorter than the ovary. Clearly, the morphological differences cited in the protologue do not provide reliable grounds for distinguishing the two taxa. Therefore, *S. neomyrtillacea* is treated here as a synonym of *S. myrtillacea*, a treatment also supported by He (2015).

The protologue of *Salix neomyrtillacea* designated *Z.D. Wei 005* as the type and stated that the type specimen was deposited at the NWBI (now HNWP). Our investigation located two specimens matching the designation: HNWP120519 and HNWP120518 (♀ only). As neither specimen bears a type designation label from Ch.Y. Yang or Z.D. Wei, both constitute syntypes. HNWP120518 contains a leafy branch (belonging to *Z.D. Wei 061*) and a pistillate flower branch (belonging to *Z.D. Wei 005*). HNWP120519 consists solely of a pistillate branch, bearing several well-preserved pistillate inflorescences. Therefore, HNWP120519 is herein designated as the lectotype of *S. neomyrtillacea*.

### 13. Salix nummularia

Taxon classificationPlantaeAplousobranchiaPolycitoridae

Andersson, Prodr. 16(2[2]): 298. 1868.

6456BB94-1558-5D3D-9342-2A57E078788F

Salix nummularia Andersson, Prodr. 16(2[2]): 298. 1868. Type: Russia, Altai, “ad fl. Tschuja”, 1832, *Bunge s.n*. (lectotype designated by Dorn (1994: 93): S-G-5406, ♀, digital image examined; isolectotype: LE barcode 01016696, digital image examined).
= Salix tschanbaischanica Y.L. Chou & Y.L. Chang, Ill. Fl. Lign. Pl. N.-E. China: 146, 557. 1955. ≡ Salix polyadenia var. tschanbaischanica (Y.L. Chou & Y.L. Chang) Y.L. Chou, Fl. Reipubl. Popularis Sin. 20(2): 275. 1984. Type: China, Liao-ning, in pratis alpinis Mt. Tschanbaischan, 2200–2400 m alt., 27 July 1950, *T.N. Liou 1560* (lectotype designated here: IFP barcode 03303002a0002!, ♂; isolectotypes: IFP barcode 03303002a0001!, IFP barcode 03303002x0008!) (Fig. 3D); ibidem, *S.G. Wang 452* (syntype, n.v.).

#### Note.

Y.L. Chou and Y.L. Chang described *S. tschanbaischanica*, based on specimens collected from Changbai Mountain (Skvortsov et al. 1955). Y.L. Chou later recombined this name as S. polyadenia var. tschanbaischanica (Wang et al. 1984). Skvortsov (1968, 1999) and Fang et al. (1999) treated the name as a synonym of *S. nummularia*. A comparison of the type specimens of *S. tschanbaischanica* and *S. nummularia* reveals no significant differences between them. Therefore, we agree with the treatment by Skvortsov (1968, 1999) and Fang et al. (1999) in considering *S. tschanbaischanica* as a synonym of *S. nummularia*.

The protologue of *S. tschanbaischanica* cited two gatherings, *T.N. Liou 1560* and *S.G. Wang 452*, but did not designate a type (only specified that the type specimen was deposited at IFP). Our investigation located three specimens matching this designation: IFP03303002a0002, IFP03303002a0001 and IFP03303002x0008, all constituting syntypes. Amongst these, IFP03303002a0002 is in the best state of preservation and bears a handwritten determination label annotated with “sp. nov.” by Y.L. Chou. Therefore, IFP03303002a0002 is herein designated as the lectotype of *S. tschanbaischanica*.

### 14. Salix psammophila

Taxon classificationPlantaeAplousobranchiaPolycitoridae

C. Wang & C.Y. Yang, Bull. Bot. Lab. N.-E. Forest. Inst., Harbin 9: 104. 1980.

F81EECF5-9237-5EF0-B55E-7FD69A0853AB

#### Type.

**Figure 4.**
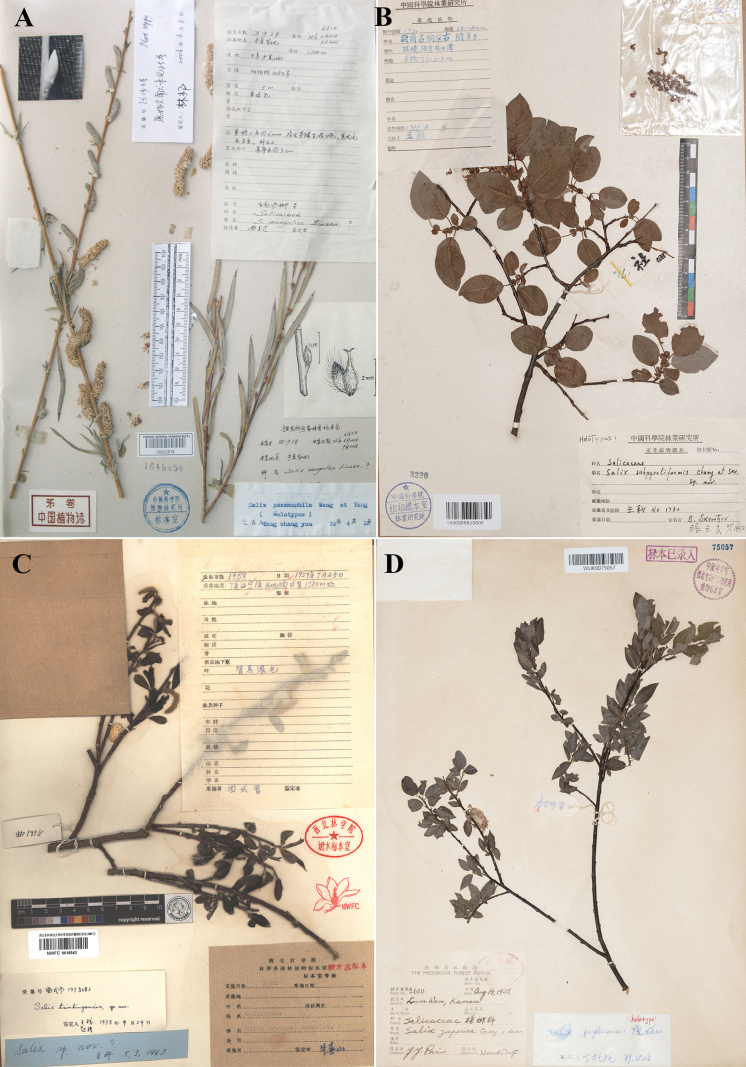
Type specimens of *Salix*. **A**. Lectotype of *S. psammophila* (left-hand fruiting branch); **B**. Lectotype of *S. subpyroliformis*; **C**. Lectotype of *S. qinlingica*; **D**. Lectotype of S. qinghaiensis var. microphylla (photo credit: **A** PE, available at https://www.cvh.ac.cn/, **B**, **D** by Zhenfeng Zhan, **C** by Xin Zhang).

China, Ningxia, Yanchi Xian, 21 May 1975, *S.K. Feng 2* (holotype, destroyed), ibidem, 21 May 1975, *S.K. Feng 4* (isotype, n.v.), ibidem, 21 May 1975, *S.K. Feng 3* (lectotype designated here: PE barcode 00023878, left-hand fruiting branch, digital image examined) (Fig. 4A); China, Shanxi, Dingbian Xian, 29 July 1972, *C.Y. Chang 17568* (paratypes, WUK barcode 0366411!, WUK barcode 0291492!, sterile); ibidem, Jingbian Xian, 10 May 1956, *C.S. Yang 1789* (paratype, WUK barcode 0081115, ♀); China, Nei Mongol, Mu Us Shamo, 1964, *S.L. Yang 29* (paratype, n.v.).

**Figure 5.**
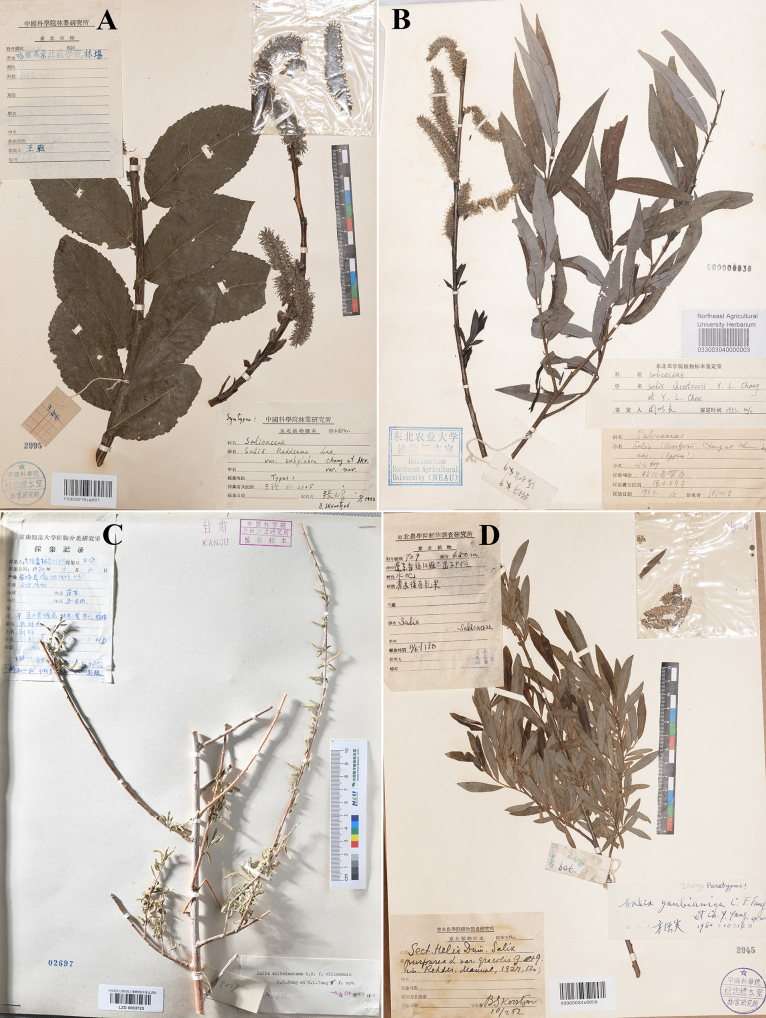
Type specimens of *Salix*. **A**. Lectotype of S. raddeana var. subglabra (left-hand branch, sterile); **B**. Lectotype of *S. skvortsovii* (left-hand branch, ♀); **C**. Lectotype of S. wilhelmsiana f. ciliuensis; **D**. Lectotype of *S. yanbianica* (photo credit: **A**, **B**, **D** by Zhenfeng Zhan, **C** LZD, available at https://www.cvh.ac.cn/).

#### Note.

C. Wang and C.Y. Yang described *S. psammophila*, based on specimens collected from Ningxia, Inner Mongolia and Shaanxi (Yang 1980). He designated “*S.K. Feng 2*” from Yanchi County, Ningxia, as the type, stating it was deposited in the Herbarium of the Ningxia Institute of Agriculture. During a field investigation in 2023, the author Zhenfeng Zhan learned from Mr. Zhu Qiang of the Ningxia Botanical Garden that the Herbarium of the Ningxia Institute of Agriculture has undergone multiple institutional reorganisations and most specimens formerly stored there, including the type of *S. psammophila*, have been lost; only a small number of specimens remain at the Ningxia Botanical Garden. The author thoroughly examined these remaining specimens and confirmed that the type of *S. psammophila* is not amongst them. After extensive searching, no duplicate specimens of “*S.K. Feng 2*” were found in any other herbarium in China. The collection information for *S.K. Feng 4* is identical to that of *S.K. Feng 2*; therefore, *S.K. Feng 4* should be considered as isotype. The author searched for *S.K. Feng 4* through CVH (https://www.cvh.ac.cn/index.php), but found no qualified specimens and, during the study at relevant herbaria, no qualified specimens were discovered either. However, while searching for specimens of *S. psammophila* through CVH, we found a specimen, PE00023878, bearing the collection number *S.K. Feng 3*, with a collection locality consistent with that of *S.K. Feng 2*. This specimen has three branches collected at different times (according to the information on the label): the female branch on the left was collected on 5 April 1975; the fruiting branch on the left was collected on 21 May 1975, exactly the same collection date as *S.K. Feng 2*; and the mature leafy branch on the right was collected on 22 September 1975. Furthermore, this specimen bears a complete identification label handwritten by C.Y. Yang, marked as “Holotype”. Clearly, the fruiting branch on the left of PE00023878, sharing the same collection locality and date as the holotype, actually constitutes a duplicate of the holotype, i.e. an isotype, even though it did not appear in the protologue of *S. psammophila* (Art. 9.5, Turland et al. (2025)). When designating the lectotype, it takes precedence over other paratypes (Art. 9.12, Turland et al. (2025)). Finally, we examined this fruiting branch: the second-year branch is yellowish-brown, with linear leaves and a very short fruiting peduncle; these morphological features are entirely consistent with the original description and the taxonomic concept of *S. psammophila*. In conclusion, we designate the fruiting branch on the left of PE00023878 as the lectotype of *S. psammophila*.

### 15. Salix pyrolifolia

Taxon classificationPlantaeAplousobranchiaPolycitoridae

Ledebour, Fl. Altaic. 4: 270. 1833.

BC0091C2-7B01-5152-8354-DF3A876FF014

Salix pyrolifolia Ledebour, Fl. Altaic. 4: 270. 1833. Type: Russia, Respublika Altay, Bunge (lectotype designated by Belyaeva (2006: 76): LE barcode 01016721, sterile, digital image examined).
= Salix subpyroliformis Y.L. Chang & Skvortsov, Ill. Fl. Lign. Pl. N.-E. China: 155, 554. 1955. Type: China, Mongolia Interior, in mountains et silvis jugi Chingan Grandis, 650–800 m alt., 4 August 1952, *Wang-Schang 1730* (lectotype designated here: IFP barcode 03303999z0006!, ♀; isolectotypes: IFP barcode 03303018x0001!, IFP barcode 03303018x0002!) (Fig. 4B).

#### Note.

Y.L. Chang and Skvortsov described *S. subpyroliformis*, based on specimens collected from Mongolia (Skvortsov et al. 1955). This name was treated as a synonym of *S. pyrolifolia* in both Flora Reipublicae Popularis Sinicae (Wang et al. 1984) and the Flora of China (Fang et al. 1999). Our comparison of the type specimens of *S. subpyroliformis* and *S. pyrolifolia* reveals no significant morphological differences between them. Therefore, we agree with the treatment by Wang et al. (1984) and Fang et al. (1999) in considering *S. subpyroliformis* a synonym of *S. pyrolifolia*.

The protologue of *S. subpyroliformis* cited *Wang-Schang 1730* and specified that the type was deposited at IFP. Our investigation located three specimens of *Wang-Schang 1730*: IFP03303999z0006, IFP03303018x0001 and IFP03303018x0002, all constituting syntypes. IFP03303018x0002 was excluded from lectotype consideration as it lacks infructescences. IFP03303999z0006 and IFP03303018x0001 are in a comparable state of preservation and both bear handwritten determination labels by Skvortsov. Therefore, IFP03303999z0006 is herein designated as the lectotype of *S. subpyroliformis*.

### 16. Salix qinlingica

Taxon classificationPlantaeAplousobranchiaPolycitoridae

C. Wang & N. Chao, Acta Bot. Boreal.-Occid. Sin. 5(2): 116. 1985.

B57D889E-3F1C-5977-9B08-18B82076ABAC

#### Type.

China, Shanxi: Ningshan Xian, Huodigou, 1780 m alt., July 1959, *S.Z. Qu 1958* (lectotype designated here: NWFC barcode 0016543!, ♀; isolectotypes: NWFC barcode 0016545!, NWFC barcode 0016542!) (Fig. 4C).

#### Note.

C. Wang and N. Chao described *S. qinlingica*, based on specimens collected from Ningshan County (Chao 1985). This name is accepted in the current taxonomic system (https://powo.science.kew.org/). The protologue designated *S.Z. Qu 1958* as the type and specified its deposition at NWFC. Our investigation located three specimens matching the designation: NWFC0016543, NWFC0016545 and NWFC0016542. None of them bears a type designation label from C. Wang or N. Chao and all constitute syntypes. As these specimens are in a similar state of preservation, NWFC0016543 is hereby designated as the lectotype of *S. qinlingica*.

### 17. Salix qinghaiensis var. microphylla

Taxon classificationPlantaeAplousobranchiaPolycitoridae

 Y.L. Chou, Bull. Bot. Res., Harbin 1(1–2): 165. 1981.

B72FE0D4-54D1-51E9-9706-F93223F98FEA

#### Type.

China, Gansu, Lunwan, 14 August 1935, *Bai Yin-yuan 2604* (lectotype designated here: WUK barcode 0075057!, ♀; isolectotype: WUK barcode 0281141!) (Fig. 4D).

#### Note.

Chou (1981) described S. qinghaiensis var. microphylla, based on specimens collected from Lunwan, Gansu. This name is accepted in the current taxonomic system (https://powo.science.kew.org/). The protologue designated *Bai Yin-yuan 2604* as the holotype and specified its deposition at the WUK Herbarium. However, our investigation located two specimens matching this designation at WUK: WUK0075057 and WUK0281141. Neither specimen bears a type designation label from Y.L. Chou and both effectively constitute syntypes. As these two specimens are in a comparable state of preservation, WUK0075057 is herein designated as the lectotype for S. qinghaiensis var. microphylla.

### 18. Salix yanbianica

Taxon classificationPlantaeAplousobranchiaPolycitoridae

C.F. Fang & C.Y. Yang, Bull. Bot. Lab. N.-E. Forest. Inst., Harbin 9: 103. 1980.

EA990531-A453-57AE-B018-832D64E1EE62

#### Type.

China, Jilin, Antu Xian, Erdaobaihe, *T.N. Liou 709* (lectotype designated here: IFP barcode 03303034x0058!, ♀; isolectotypes: IFP barcode 03303034x0057!, IFP barcode 03303034x0059!, IFP barcode 03303034z0001!) (Fig. 5D). Remaining syntypes: China, Jilin, Antu Xian, Erdaobaihe, *T.N. Liou 791* (IFP barcode 03303034x0064!, IFP barcode 03303034x0063!, IFP barcode 03303034x0062!, IFP barcode 03303034x0061!, IFP barcode 03303034x0060!, ♀).

#### Note.

C.F. Fang and C.Y. Yang described *S. yanbianica*, based on specimens collected from Antu County, Jilin (Yang 1980). This name is accepted in the current taxonomic system (https://powo.science.kew.org/). The protologue designated *T.N. Liou 709* as the type and stated that the type specimen was deposited at IFP. Our investigation located four specimens matching this designation: IFP03303034x0058, IFP03303034x0057, IFP03303034x0059 and IFP03303034z0001. All bear a complete handwritten identification label with the notation “Paratype” by C.F. Fang and all constitute syntypes. Amongst these, IFP03303034x0058 is well-preserved, bearing numerous infructescences and a complete handwritten determination label by C.F. Fang. Therefore, IFP03303034x0058 is herein designated as the lectotype for *S. yanbianica*.

## Supplementary Material

XML Treatment for Salix babylonica

XML Treatment for Salix bebbiana

XML Treatment for Salix bikouensis var. villosa

XML Treatment for Salix caprea

XML Treatment for Salix cyanolimenaea

XML Treatment for Salix gordejevii

XML Treatment for Salix juparica

XML Treatment for Salix juparica var. tibetica

XML Treatment for Salix kangensis

XML Treatment for Salix matsudana

XML Treatment for Salix miyabeana

XML Treatment for Salix myrtillacea

XML Treatment for Salix nummularia

XML Treatment for Salix psammophila

XML Treatment for Salix pyrolifolia

XML Treatment for Salix qinlingica

XML Treatment for Salix qinghaiensis var. microphylla

XML Treatment for Salix yanbianica

## Additional information

### Artificial Intelligence (AI) use

The authors accept full responsibility for the content of the manuscript, including the disclosure of any use of AI.

No AI tools were used in the preparation of this manuscript.

### Author ORCIDs

Zhenfeng Zhan https://orcid.org/0009-0001-3319-613X

Hong Qu https://orcid.org/0009-0003-5662-3434

Xin Zhang https://orcid.org/0000-0003-0669-8342

Zhangjie Huang https://orcid.org/0000-0002-2288-6952

Changli Zhao https://orcid.org/0009-0008-7724-7841

Lei Wang https://orcid.org/0000-0002-1125-567X

### Data availability

All of the data that support the findings of this study are available in the main text.

## References

[B1] Andersson NJ (1860) On East Indian Salices. Journal of the Linnean Society, Botany 4: 49–58.

[B2] Andersson NJ (1868) Salicineae. In: Candolle A de (Ed.), Prodromus Systematis Naturalis Regni Vegetabilis, vol. 16(2). Treuttel et Würtz, Paris, 191–324.

[B3] Argus GW (1986) The genus *Salix* (Salicaceae) in the southeastern United States. Systematic botany monographs 9: 1–170. 10.2307/25027618

[B4] Argus GW (1997) Infrageneric classification of *Salix* (Salicaceae) in the New World. Systematic Botany Monographs 52: 1–121. 10.2307/25096638

[B5] Belyaeva IV, Epanchintseva OV, Shatalina AA, Semkina LA (2006) Willows of Ural: Atlas and identification key. Russian Academy of Sciences, Ekaterinburg, 1–173.

[B6] Belyaeva IV, King C (2025) 1138. *Salix magnifica* Hemsl.: Salicaceae. Botanical Magazine 42(1): 77–94. 10.1111/curt.12631

[B7] Chang YL, Chou YL (1955) Salicaceae. In: Chou YL, Huang LZ, Chang YL, Li QT, Zhao DC (Eds), Woody Plants of the Lesser Khingian Mountains. China Forestry Publishing House, Beijing, 73–91.

[B8] Chao N (1985) New taxa of *Salix* L. from Shaanxi. Acta Botanica Boreali-Occidentalia Sinica 5(2): 115–117.

[B9] Chou YL (1981) Plantae novae Salicium Sinicae. Bulletin of botanical research 1(2): 159–166.

[B10] Ding TY (1995) Origin, divergence and geographical distribution of Salicaceae. Acta Botanica Yunnanica 17(3): 277–290.

[B11] Dorn RD (1994) North American *Salix* (Salicaceae) typifications and notes. Phytologia 77(2): 89–95.

[B12] Fang CF (1984) Taxa nova Salicaearum e China boreali-orientali. Bulletin of botanical research 4(1): 123–127.

[B13] Fang CF, Zhao SD, Skvortsov AK (1999) Salicaceae, *Salix*. In: Wu ZY & Raven PH (Eds) Flora of China, vol. 4. Science Press, Beijing & Missouri Botanical Garden, St. Louis, 162–274.

[B14] Goerz R (1932) Enumeration of the Ligneous Plants Collected by J.F. Rock on the Arnold Arboretum Expedition to Northwestern China and Northeastern Tibet, *Salix* L. Journal of the Arnold Arboretum 13(4): 387–404.

[B15] Govaerts R, Nic Lughadha E, Black N, Turner R, Paton A (2021) The World Checklist of Vascular Plants, a continuously updated resource for exploring global plant diversity. Scientific Data 8: e215. 10.1038/s41597-021-00997-6 PMC8363670 34389730

[B16] Grabovskaya-Borodina AE, Tatanov IV, Belyaeva IV (2022) Nomenclatural and taxonomic notes on some *Salix* L. (Salicaceae) from China. Skvortsovia: International Journal of Salicology and Plant Biology 8(2): 58–81. 10.51776/2309-6500_2022_8_2_58

[B17] Hao KS (1936) Synopsis of Chinese *Salix*. Repertorium Specierum Novarum Regni Vegetabilis, Beihefte 93: 1–123.

[B18] He L (2015) Taxonomic Revision of *Salix* L. (Salicaceae) in Pan Himalayas. PhD Thesis, Beijing Forestry University, China.

[B19] He L, Liao S, Applequist W, Chen SP (2019) The valid publication of *Salix suchowensis* (Salicaceae). PhytoKeys 131: 27–35. 10.3897/phytokeys.131.37065 PMC6733804 31537961

[B20] He L, Wagner ND, Hörandl E (2021) Restriction‐site associated DNA sequencing data reveal a radiation of willow species (*Salix* L., Salicaceae) in the Hengduan Mountains and adjacent areas. Journal of Systematics and Evolution 59(1): 44–57. 10.1111/jse.12593

[B21] Jonsell B, Jarvis CE (1994) Lectotypifications of Linnaean names for Flora Nordica, vol. 1 (Lycopodiaceae–Papaveraceae). Nordic Journal of Botany 14(2): 145–164. 10.1111/j.1756-1051.1994.tb00581.x

[B22] Jordaan M (2005) FSA contributions 18: Salicaceae s. str. Bothalia 35(1): 7–20. 10.4102/abc.v35i1.364

[B23] Koidzumi G (1915) Decades Plantarum Novarum vel minus Cognitarum. Botanical Magazine (Tokyo) 29: 309–332. 10.15281/jplantres1887.29.348_309

[B24] Linnaeus C (1753) Species Plantarum. Impensis Laurentii Salvii, Holmiae, 1–1200.

[B25] Liu LJ, He L, Applequist WL (2020) Untangling two Chinese *Salix* species (Salicaceae) published by C.K. Schneider, with lectotypification of four names. Willdenowia 50(2): 159–163. 10.3372/wi.50.50201

[B26] Ohashi H (2000) A systematic enumeration of Japanese *Salix* (Salicaceae). Journal of Japanese Botany 75(1): 1–41.

[B27] Ohashi H (2001) Salicaceae of Japan. Science Reports of the Tohoku Imperial University, series 4, Biology 40(4): 269–396.

[B28] Sargent CS (1895) The Names of some North American Tree Willows. Garden & Forest 8(404): 463. 10.5281/zenodo.3929906

[B29] Seemen OV (1896) Neue Weidenarten in dem Herbar des Königlichen botanischen Museums zu Berlin II. Botanische Jahrbücher für Systematik 21(4): 50–58.

[B30] Skvortsov AK, Chang YL, Chou YL, Wang C (1955) Salicaceae. In: Liu SE (Ed.) Illustrated Manual of the Woody Plants of the Northeast Province. Science Press, Beijing, 110–187.

[B31] Skvortsov AK (1968) Willows of the USSR. A taxonomic and geographic revision. Nauka, Moscow.

[B32] Skvortsov AK (1989) Die Weiden (*Salix*) der Sektion *Chamaetia* und das Problem der Entstehung der arktischen Flora. Flora 182: 57–67. 10.1016/s0367-2530(17)30394-8

[B33] Skvortsov AK (1999) Willows of Russia and adjacent countries. Taxonomical and geographical revision. Faculty of Mathematics and Natural Sciences Report Series, No. 39. University of Joensuu Press, Joensuu, 1–307.

[B34] Thiers B (2025) Index Herbariorum: A global directory of public herbaria and associated staff. New York Botanical Garden’s Virtual Herbarium. https://sweetgum.nybg.org/science/ih/

[B35] Trautvetter ER (1833) *Salix*. In: Ledebour CF (Ed.) Flora Altaica, vol. 4. Typis et impensis G. Reimeri, Berolini, 251–292.

[B36] Turland NJ, Wiersema JH, Barrie FR, Gandhi KN, Gravendyck J, Greuter W, Hawksworth DL, Herendeen PS, Klopper RR, Knapp S, Kusber W-H, Li D-Z, May TW, Monro AM, Prado J, Price MJ, Smith GF, Zamora Senioret JC (2025) International Code of Nomenclature for algae, fungi, and plants (Madrid Code). Regnum Vegetabile 162. University of Chicago Press, Chicago. 10.7208/chicago/9780226839479.001.0001

[B37] Wang Z, Fang CF, Zhao SD, Chou YL, Dong SL, Yu CY, Yang CY, Chao N (1984) *Salix*. In: Wang C & Fang CF (Eds) Flora Reipublicae Popularis Sinicae, vol. 20(2). Science Press, Beijing, 81–381.

[B38] Wei ZD (1987) Salicaceae, *Salix*. In: Li YJ (Ed.) Woody Flora of Qinghai. Qinghai People’s Publishing House, Xining, 81–138.

[B39] Yan JZ, Fang CF, Qin ZS (1981) A new variety of *Salix*. Bulletin of botanical research 1(2): 175–178.

[B40] Yang CY (1980) Plantae novae Salicum Sinicae (IV). Bulletin of the Botanical Laboratory of the North-Eastern Forestry Institute 9: 89–107.

[B41] Yang XL (1985) Addenda: diagnoses taxorum novarum, vol. 2. In: Liu YX (Ed.) Flora in desertis Reipublicae Populorum Sinarum. Science Press, Beijing, 521–522.

